# Detailed Insight into the Interaction of Bicyclic Somatostatin Analogue with Cu(II) Ions

**DOI:** 10.3390/ijms21228794

**Published:** 2020-11-20

**Authors:** Aleksandra Marciniak, Weronika Witak, Giuseppina Sabatino, Anna Maria Papini, Justyna Brasuń

**Affiliations:** 1 Department of Inorganic Chemistry, Wroclaw Medical University, Borowska 211A, 50-556 Wroclaw, Poland; aleksandra.marciniak@umed.wroc.pl (A.M.); weronika.witak@umed.wroc.pl (W.W.); 2 Interdepartmental Research Unit of Peptide and Protein Chemistry and Biology, Department of Chemistry “Ugo Schiff”, University of Florence, Via della Lastruccia 13, 50019 Sesto Fiorentino, Italy; giuseppina.sabatino@cnr.it (G.S.); annamaria.papini@unifi.it (A.M.P.); 3 CNR-IC Istituto di Cristallografia, Via Paolo Gaifami 18, 95126 Catania, Italy

**Keywords:** somatostatin analogues, bicyclic peptide, copper(II) complexes, potentiometric titration, magnetic circular dichroism

## Abstract

Somatostatin analogues are useful pharmaceuticals in peptide receptor radionuclide therapy. In previous studies, we analyzed a new bicyclic somatostatin analogue (BCS) in connection with Cu(II) ions. Two characteristic sites were present in the peptide chain: the receptor- and the metal-binding site. We have already shown that this ligand can form very stable imidazole complexes with the metal ion. In this work, our aim was to characterize the intramolecular interaction that occurs in the peptide molecule. Therefore, we analyzed the coordination abilities of two cyclic ligands, i.e., P1 only with the metal binding site and P2 with both sites, but without the disulfide bond. Furthermore, we used magnetic circular dichroism (MCD) spectroscopy to better understand the coordination process. We applied this method to analyze spectra of P1, P2, and BCS, which we have described previously. Additionally, we analyzed the MCD spectra of P3 ligand, which has only the receptor binding site in its structure. We have unequivocally shown that the presence of the Phe-Trp-Lys-Thr motif and the disulfide bond significantly increases the metal binding efficiency.

## 1. Introduction

**Figure 1:**
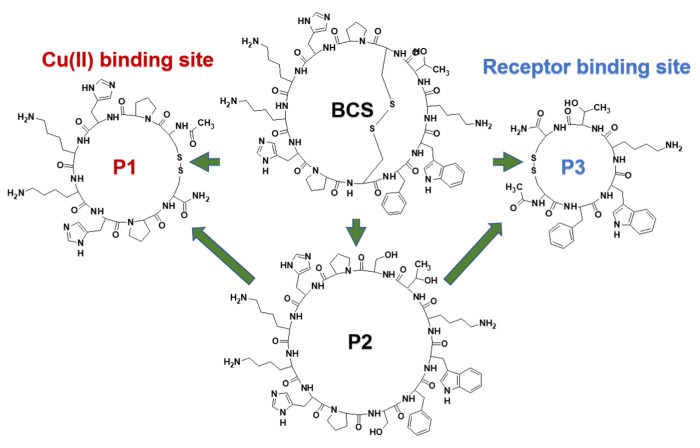
Structures of the bicyclic somatostatin analogue (BCS) (c(c(-S-Cys-**Phe-Trp-Lys-Thr-**Cys-S-)Pro-**His-Lys-Lys-His-**Pro)) [13] and the three designed peptides that were analyzed: P1. (Ac-c(-S-Cys-Pro-His^a^-Lys-Lys-His^b^-Pro-Cys-S-)-NH_2_); P2. (c(Ser-Pro-His^a^-Lys-Lys-His^b^-Pro-Ser-Phe-Trp-Lys-Thr); and P3. Ac-c(-S-Cys-Phe-Trp-Lys-Thr-Cys-S)-NH_2_.

Bicyclic peptides are very interesting lead compounds that are used in medical and pharmaceutical applications. Their properties make them attractive and promising tools in the field of nuclear medicine [1]. Among the bicyclic peptides described in the literature, there are a number of reports on somatostatin (SST) analogues [2,3,4,5,6,7]. SST derivatives play an important role in the diagnosis and treatment of neuroendocrine tumors [8] and their complexes with radioactive isotopes are already used in peptide receptor radionuclide therapy [9,10,11,12]. In our previous studies, we have described the ability of bicyclic somatostatin analogue (BCS) to coordinate with the sequence c(c(-S-Cys-**Phe-Trp-Lys-Thr-**Cys-S-)Pro-**His-Lys-Lys-His-**Pro) (Figure 1) [13]. Potentiometric and spectroscopic analyses have shown that BCS is an effective ligand for Cu(II) ions and its coordinating abilities are different from simple cyclopeptides. During the coordination process Cu(II) forms a series of mononuclear complexes and only imidazole donors are involved below pH 7. The complexes with only imidazole donors in BCS are characterized by high stability, which is significantly higher in comparison to other peptides. This effect may be caused by certain intramolecular interactions, e.g., with Trp or Phe side-chains located in the receptor binding site; however, this phenomenon is still unexplained. Therefore, we decided to analyze new peptide sequences and confirm our assumptions.

In particular, in this paper we have focused on the coordination abilities of two monocyclic peptides:

P1. *Ac-c(-S-Cys-Pro-**His^a-^Lys-Lys-His^b^-**Pro-Cys-S-)-NH_2_*
P2. *c(Ser-Pro-**His^a^-Lys-Lys-His^b^-**Pro-Ser-**Phe-Trp-Lys-Thr**)* (Figure 1).

Previous analyzed BCS showed two characteristic peptide fragments: the metal binding site with two histidine residues and the receptor binding site **Phe-Trp-Lys-Thr**, which is also present in the native hormone and responsible for interaction with receptors. Herein, we focused our attention on the analysis of Cu(II) interactions with the metal binding cycle of the ligand and the influence of the receptor binding site on the coordination process. Therefore, both P1 and P2 were designed to contain a Pro-**His^a^-Lys-Lys-His^b^**-Pro sequence, whereas the receptor binding site **Phe-Trp-Lys-Thr** was only introduced in P2. Proline residues are “break points” and stop coordination with subsequent amide nitrogen atoms in the peptide chain [14]. Therefore, the place where the metal ion can be coordinated is better defined. The somatostatin derivatives we designed to be synthesized and analyzed contain elements of novelty in comparison to the SST analogues that are currently used in medicine, where the metal ion is coordinated by chelators such as 1,4,7,10-tetraazacyclododecane-1,4,7,10-tetraacetic acid (DOTA) and linked to bioactive peptide molecules via a linker. The analysis of the binding abilities of P1 and P2 may help us to understand the role of the aromatic side chains and how the receptor binding site influences the coordination abilities of the previously studied BCS. Potentiometric titrations, UV-Vis, and circular dichroism (CD) spectroscopies were used herein. Furthermore, we applied a magnetic circular dichroism spectroscopy (MCD) to obtain a better understanding of the role of Phe and Trp aromatic side-chains,. We used this method for peptides BCS, P1, P2, and P3 with the sequence Ac-c(-S-Cys-**Phe-Trp-Lys-Thr**-Cys-S)-NH_2_. The experiments that we performed allowed us to investigate any interactions and details that may have an impact on the coordination properties of the analyzed peptides.

## 2. Results and Discussion

Table 1 shows the acid-base properties of the ligands and the stability constants of the complexes formed with Cu(II) ions. Both ligands, P1 and P2, have four or five side-chain functionalities in their sequences that are prone to deprotonation: two (in the case of P1) or three (in the case of P2) amino groups of the lysine residues and two imidazole rings of the two histidine residues. Both peptides P1 and P2 show similar acid base properties. Potentiometric analyses clearly showed that in both cases, it was possible to obtain four protonation constants: two of them that are characteristic for His residues (≈5 and ≈6) and two for Lys (≈10 and ≈9). In the experimental conditions, the calculation of the protonation constant of one of the Lys residues in the case of P2 was not possible.

**Figure 2:**
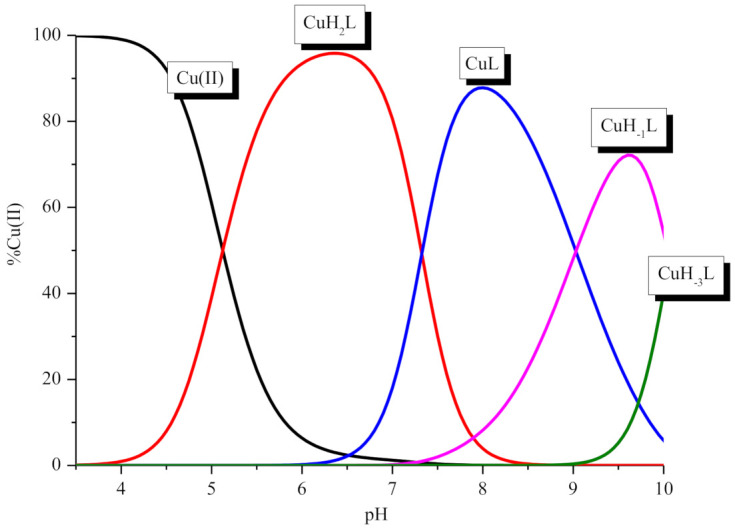
Species distribution curves for the Cu(II)/P1 system in relation to the pH. The ligand concentration was 7 × 10^−4^ mol/L and pH-metric titration was performed in a 0.3 mol/L KCl solution using sample volumes of 1.5 mL. Measurements were carried out in a 2.5–10 pH range, at 25 °C. The ligand to metal ratio was 2:1.

The sequence of P1 corresponds to the metal binding site in P2. Thus, we first focused on P1. Analysis of the potentiometric data shows that the ligand forms four complexes between pH 2.5 and 10 (Table 1, Figure 2). The metal coordination starts above pH 4 and forms the CuH_2_L species, which achieves its highest concentration around pH 6.5 (Figure 2). The appearance of this complex is related to the loss of two protons in the peptide molecule. The corrected logβ*_CuH2L_ = log*β_CuH2L_* − log*β_H2L_* = 4.97 is relatively low for a complex with two imidazole donors involved in Cu(II) coordination [15]. However, the location of the d-d band at 665 nm (Table 2) supports the presence of two nitrogen atoms in the coordination sphere of the Cu(II) ion [16].

**Figure 3:**
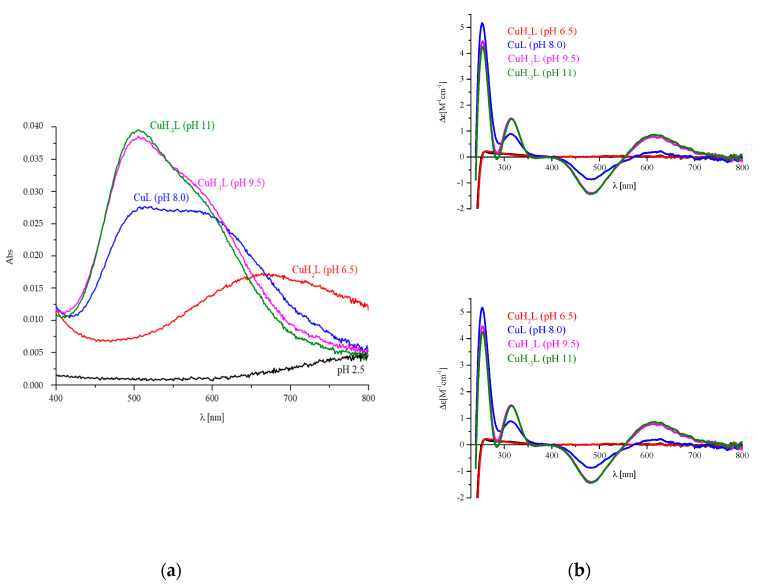
Spectra for Cu(II)/P1 system: (**a**) UV-Vis; (**b**) circular dichroism (CD). The ligand concentration was 7 × 10^−4^ mol/L and pH-metric titration was performed in a 0.3 mol/L KCl solution using sample volumes of 2 mL. Measurements were carried out in a 2.5–10 pH range, at 25 °C. The pH values were established by adding small amounts of concentrated KOH and HCl solutions. The ligand to metal ratio was 2:1.

The simultaneous dissociation of two protons was observed when there was an increase in pH, and the CuL complex was formed. Its appearance in the solution significantly influences the spectral abilities of the system. The blue shift in the λ_max_ for d-d transition 665 nm → 500 nm (Figure 3a) strongly suggests the involvement of the next nitrogen atoms in metal ion binding. Moreover, the appearance of the positive CT transition in CD spectrum at 315 nm (Figure 3b, Table 2) shows the presence of the amide donor in the coordination sphere of Cu(II).

At pH 9, the CuH_−1_L species dominates the system. Its formation also influences the spectral abilities of the solution. The increase in the ∆ε_315_ supports the involvement of the next amide donor and the formation of the square planar complex with the {N_Im_, 3N_amide_} binding mode.

Finally, the CuH_−3_L species appears in the system. However, its presence has no significant impact on the spectral abilities. Moreover, the log*K*_CuH−1L_ → _CuH−3L_ = 20.12 is comparable to the sum of log*K’s* of both Lys residues (10.29 + 9.78 = 20.07) (Table 1).

**Figure 4:**
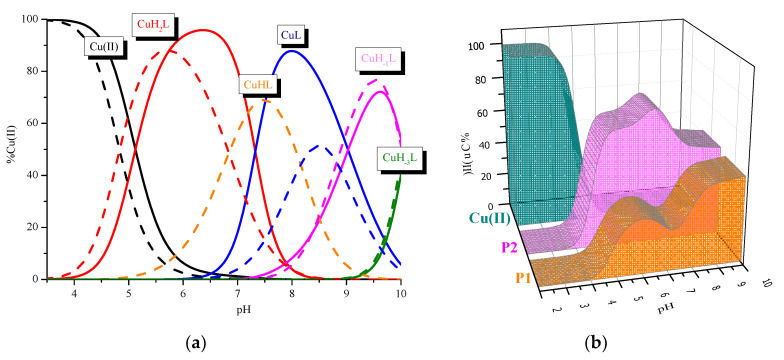
(**a**) Comparison of species distribution curves between Cu(II)/P1 (solid line) and Cu(II)/P2 (dashed line), (**b**) Comparison of the P1/Cu(II)/P2 systems. The ligands concentrations were 7 × 10^−4^ mol/L and pH-metric titrations were performed in a 0.3 mol/L KCl solution using sample volumes of 1.5 mL. Measurements were carried out in a 2.5–10 pH range, at 25 °C. The ligand to metal ratio was 2:1.

A comparison of the binding abilities of P1 and P2 (Figure 4, Table 1) allows us to characterize the role of the Phe-Trp-Lys-Thr motif in the Cu(II) binding site by both peptides.

**Figure 5:**
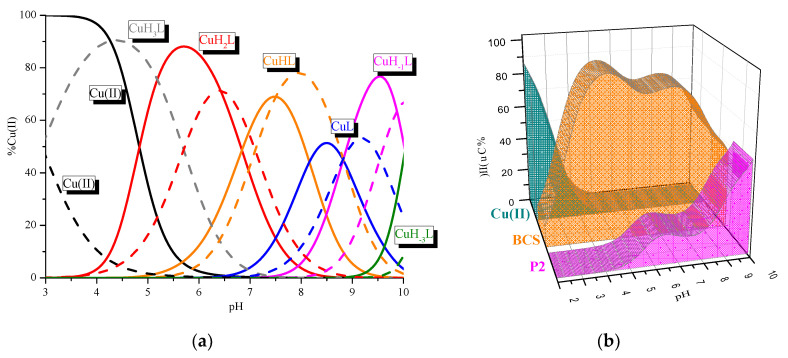
(**a**) Comparison of (a) species distribution curves for Cu(II)/P2 (solid line) and Cu(II)/BCS (dashed line) systems in relation to pH; (**b**) Comparison of the P2/Cu(II)/BCS systems. P2 concentration was 7 × 10^−4^ mol/L and pH-metric titration was performed in a 0.3 mol/L KCl solution using sample volumes of 1.5 mL. Measurements were carried out in a 2.5–10 pH range, at 25 °C. The ligand to metal ratio was 2:1.

P2 forms complexes similar to the P1 ligand. However there are some differences: (i) the complex CuH_2_L with only imidazole donors is significantly more stable (higher value of logβ*_CuH2L_ = 5.29); (ii) P2 creates CuHL species with one amide donors, which was not observed in the system with the P1 peptide; (iii) the involvement of the second amide donor (CuL complex) can be observed in significantly more basic conditions; and (iv) the formation of the species with three amide donors (CuH_−1_L) takes place in the same range of pH in both systems. Moreover, a comparison between both ligands with respect to the efficiency in Cu(II) binding (Figure 4b) shows that in the whole range of pH, P2 is definitely more effective in copper (II) binding. The analysis of both peptide structures shows that due to the presence of Pro residues, there is only one opportunity to involve the amide donors: between both His residues. The results presented above clearly show that the presence of the receptor binding site influences the binding abilities of the ligand. Above all, it significantly increases the efficiency of metal ion coordination. Due to this fact, the coordinating abilities of BCS and P2 were also compared (Figure 5). Both compounds form the same dominant complexes. However, the presence of the disulfide bond in the BCS structure influences the stability of selected species and the efficiency of metal ion binding. Firstly, BCS forms a very stable CuH_3_L complex with one imidazole donor involved in Cu(II) binding and formation of the species CuH_2_L with the {2xN_Im_} was observed in more basic conditions. Secondly, the complex with the {2N_Im_, N_amide_} coordination mode is more stable, and finally, BCS is significantly more efficient in the analyzed range of pH (Figure 5b).

**Figure 6:**
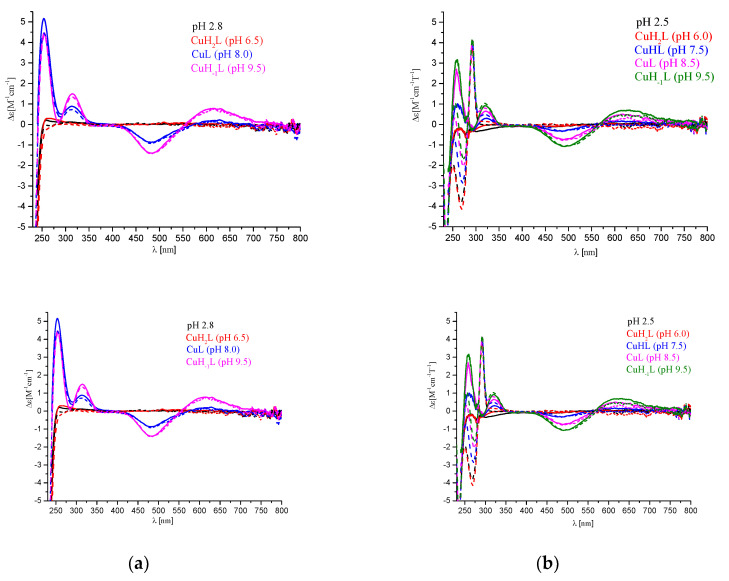
Comparison of CD (solid line) and magnetic circular dichroism (MCD) (dashed line) spectra for: (**a**) the Cu(II)/P1 system; (**b**) the Cu(II)/P2 system. The ligand concentrations were 7 × 10^−4^ mol/L and pH-metric titrations were performed in a 0.3 mol/L KCl solution. Sample volumes were 2.0 mL. The pH values were established by adding small amounts of concentrated KOH and HCl solutions.

A magnetic circular dichroism spectroscopy (MCD) was applied to achieve a better understanding of the differences in the coordination abilities of all the peptides. Figure 6 shows the comparison of the CD and MCD spectra obtained for P1 (Figure 6a) and P2 (Figure 6b). The application of the magnetic field does not influence the region where the d-d transitions are observed (above 500 nm). Therefore, the analysis of the region below 350 nm was performed in relation to the spectra of free ligands.

**Figure 7:**
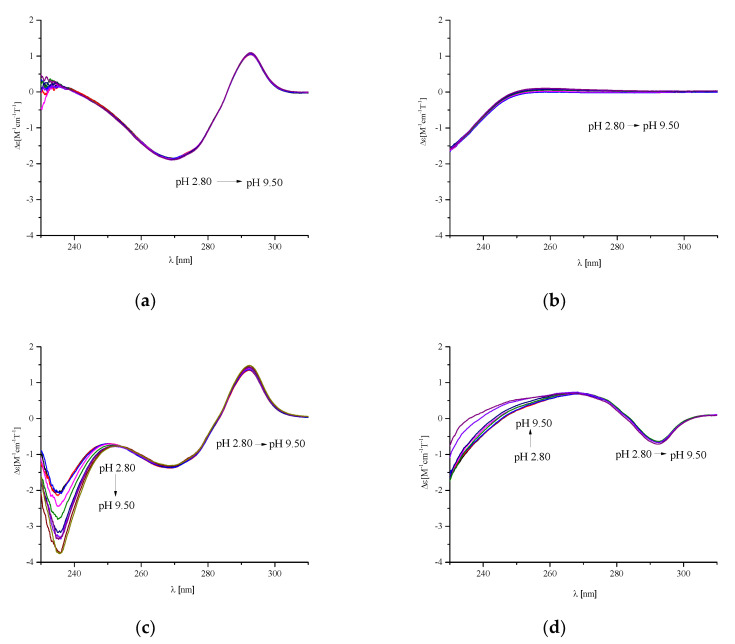
pH-dependent MCD spectra of: (**a**) P3: Ac-c(-S-Cys-Phe-Trp-Lys-Thr-Cys-S)-NH_2_; (**b**) P1: Ac-c(-S-Cys-Pro-His-Lys-Lys-His-Pro-Cys-S-)-NH_2_; (**c**) P2: c(Ser-Phe-Trp-Lys-Thr-Ser-Pro-His-Lys-Lys-His-Pro); (**d**) BCS c(c(-S-Cys-Phe-Trp-Lys-Thr-Cys-S)-Pro-His-Lys-Lys-His-Pro). The ligand concentration was 7 × 10^−4^ mol/L and pH-metric titrations were performed in a 0.3 mol/L KCl solution. Sample volumes were 2.0 mL. The pH values were established by adding small amounts of concentrated KOH and HCl solutions.

Figure 7 shows the comparison of the pH-depended MCD spectra obtained for P1, P2, and BSC (Figure 7b–d) in relation to the MCD spectrum of the P3 ligand (Ac-c(-S-Cys-**Phe-Trp-Lys-Thr-Cys**-S-)-NH_2_ sequence, Figure 7a). It can be observed that pH does not influence the MCD spectrum of P3, where two sharp intra-ligand transitions are observed at 269 nm and 293 nm with |∆ε| ≈ 3 and 2, respectively, and two shoulders at 273 nm and 283 nm (Figure 7a); these are characteristic of Trp residue [17,18]. In the P3 structure, two additional two chromophores are also present: Phe and disulfide bond, but in the region discussed they have weak bands and they are not present in the spectrum of the analyzed ligand [19]. In the MCD spectra of P2 and BCS, the pH-independent transitions that are characteristic of Trp residues can be seen, as was also the case in the P3 spectra. The pH-dependent difference, observed below 268 nm, may be caused by the presence of two His residues in P2 and BCS molecules.

**Figure 8:**
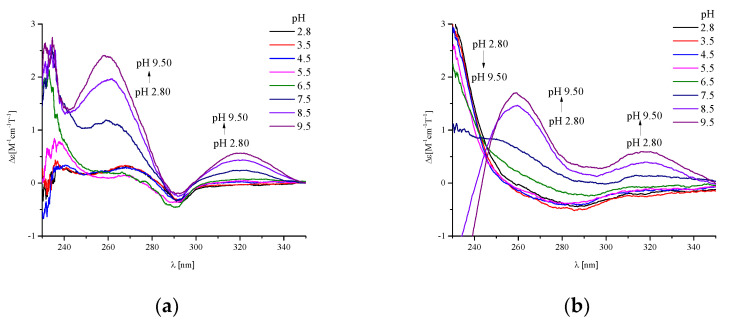

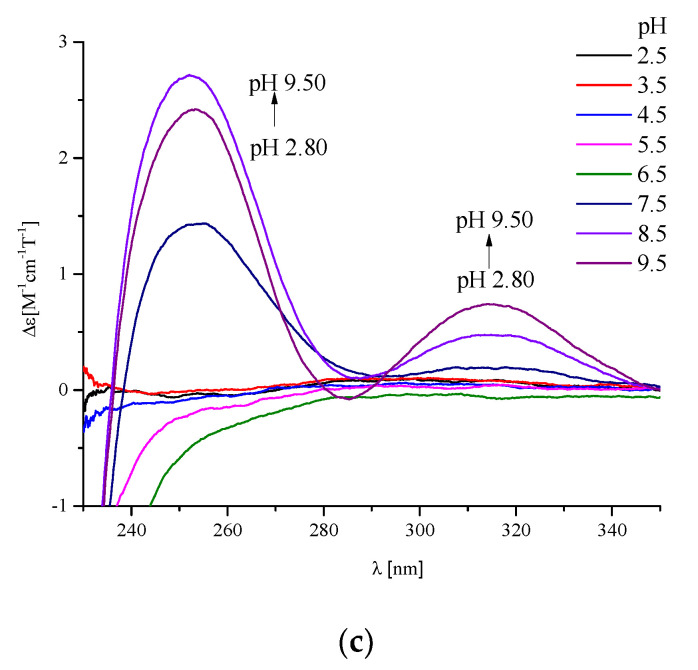
pH-dependent MCD corrected spectra of the systems: (**a**) Cu(II)/P2-P2_free;_ (**b**) Cu(II)/BCS-BCS_free,_ (**c**) Cu(II)/P1-P1_free_. The ligand concentration was 7 × 10^−4^ mol/L and pH-metric titrations were performed in a 0.3 mol/L KCl solution. Sample volumes were 2.0 mL. The pH values were established by adding small amounts of concentrated KOH and HCl solutions.

Then, we analyzed the MCD spectra of both the Cu(II)/P2 and Cu(II)/BCS systems, corrected of the free ligand (the ligand spectrum was subtracted from the complex spectrum and the result was converted to Δε [M^-1^ cm^-1^T^-1^], see Figure 8a,b).

The main difference between the BCS and P2 structures is the presence of two cycles that are created as a result of the disulfide bond between the cysteinyl moieties in the BCS peptide, and which has a strong impact on the coordination process.

The potentiometric studies showed that in the analyzed Cu(II)/P1, Cu(II)/P2, and in the previously described Cu(II)/BCS systems, the interactions of the metal ion with side chains of His residues dominated below pH 6.5–7.5, while, coordination of the amide donors was observed above pH 7.5. Analysis of the MCD results (Figure 8) showed that metal ion coordination did not have a significant influence on the band associated with tryptophan. Therefore, it can be concluded that this fragment of the peptide molecule has no major influence on the binding of the metal ion. Spectra for all three analyzed ligands were similar between 240–340 nm. However, below 240 nm, the spectra for BCS were significantly different from the other two peptides. Therefore, it seems that the presence of a double-cyclic structure determines the high efficiency of BCS, probably due to the formation of the intra-molecular hydrogen bond. The arrangement of atoms seems to be highly ordered in a molecule constructed in this way, which significantly affects the process of metal ion coordination. The access of the metal ion to the donors from the side chain groups is thus facilitated, which increases the effectiveness of Cu(II) coordination.

## 3. Materials and Methods

### 3.1. Synthesis of the Cyclopeptide C(Ser-Pro-His-Lys-Lys-His-Pro-Ser-Phe-Trp-Lys-Thr) (P2)

The head-to-tail cyclopeptide P2, was obtained via cyclization in a solution of the linear precursor, H-Trp-Lys(Dde)-Thr-Ser-Pro-His-Lys(Dde)-Lys(Dde)-His-Pro-Ser-Phe-OH, obtained in the solid-phase and cleaved from the resin partially protected by the amino functions on the side-chains of the Lys residues as N-1-(4,4-dimethyl-2,6-dioxocyclohex-1-ylidene)ethyl group (Dde). The synthesis in the solid-phase of the linear precursor was performed with an automated CEM Liberty Blue^TM^ peptide synthesizer, equipped with a Discovery^®^ microwave (MW) reactor (Matthews, NC, USA). The fully automated MW-assisted peptide synthesis (MW-SPPS) was performed following the Fmoc/tBu strategy, starting from Fmoc-Phe-Wang resin (loading 0.59 mmol/g, 170 mg, 0.1 mmol), and after deprotection of the Fmoc group, the following amino acids were adequately orthogonally protected and were added from the C- to the N-terminal: Fmoc-Ser(tBu)-OH, Fmoc-Pro-OH, Fmoc-His(Trt)-OH, Fmoc-Lys(Dde)-OH, Fmoc-Thr(tBu)-OH, Fmoc-Trp(Boc)-OH, in the presence of the coupling reagents Oxyma pure and DIC.

Reaction temperatures were monitored by an internal fiberoptic sensor. Both deprotection and coupling reactions were performed in a Teflon vessel under microwave energy and nitrogen bubbling. The Fmoc/tBu MW-SPPS protocol consisted of: (1) swelling in DMF for 30 min; (2) double deprotection (20% piperidine in DMF) at (a) 15 sec, 75 °C, 155 W; (b) 50 sec, 90 °C, 30 W; (3) three washes with DMF; (4) coupling with (a) protected amino acids as described above (5 eq, 0.2 M in DMF), and (b) addition of the coupling reagents Oxyma pure (5 eq, 1M in DMF) and DIC (5 eq, 0.5M in DMF) in separate bottles; and (5) washing with DMF (3 × 5 mL). Peptide elongation was performed by repeating the general cycle for each amino acid. After deprotection of the Fmoc group from the last amino acid, the resin was filtered, washed with DMF (3 × 5 mL) and 2-propanol (3 × 5 mL) and dried under vacuum. The cleavage, with concomitant deprotection of acid labile amino-acid side-chains, was achieved by treatment of the peptide-resin with TFA/TIS/H_2_O (10 mL, 95/2.5/2.5). The cleavage was carried out for 2.5 h at room temperature under magnetic stirring. The resin was filtered and rinsed with fresh TFA. The cleavage mixture was precipitated by the addition of ice-cold Et_2_O (20 mL). The precipitated crude peptide was washed with ice-cold Et_2_O (4 × 25 mL) and dried under vacuum. The head-to-tail cyclization of the crude peptide was performed in solution by PyBop (6 eq) and DIPEA (6 eq) in DMF (1 mg crude peptide in 10 mL 0.5 mM DMF). After obtaining the head-to-tail cyclopeptide P2, the Dde protecting group on the Lys side-chains were removed with a fresh solution of 2% N_2_H_4_ in DMF (2 × 10 min) and washed with DMF and DCM. Both lactam formation and Dde removal were monitored by HPLC-MS. Final purification of peptide P2 was carried out by semi-preparative HPLC (model 600, Waters, Milford, MA, USA) using a Phenomenex Jupiter C18 column (180Å, 5μm, 250 × 10 mm); flow: 4 mL/min; eluents: 0.1% TFA in H_2_O (A) and 0.1% TFA in CH_3_CN (B); gradient: 20–80% B in 30 min. Characterization of peptide P2 was performed by HPLC–ESI–MS (Waters Alliance 2695, Separations Module 2996 PDA coupled to Micromass ZQ) using a Phenomenex Kinetex^TM^ C18 column (100Å, 2.6 µm, 100 × 3 mm, Torrance, CA, USA); flow: 0.6 mL/min; eluents: 0.1% TFA in H_2_O (A) and 0.1% TFA in 84% CH_3_CN/H_2_O (B); gradient: 20–80% B in 20 min. After purification we obtained the cyclopeptide P2 (13 mg, 8.9% yield, 90% HPLC purity); Rt: 3.16 min; ESI-MS (m/z): [M + H]^+^: 1462.02 (found); 1461.77 (calcd.). Peptides P1 and P3 were purchased from STI (Poland, Poznań).

### 3.2. Potentiometric Measurements

Potentiometric measurements were carried out using the Metrohm (Switzerland, Herisau) pH-meter system with a Methrom semi-micro combination electrode at 25 °C, calibrated in a hydrogen ion concentration using HCl [20]. The ligand concentrations were 7 × 10^−4^ mol/L and pH-metric titrations were performed in a 0.3 mol/L KCl solution, using sample volumes of 1.5 mL. Potassium hydroxide (KOH) was added with a 2 mL micrometer syringe. The KOH concentration was 0.1 mol/L. Measurements were carried out in the 2.5–10 pH range. The stability constants and stoichiometry of the complexes were calculated from the titration curves using HYPERQUAD 2008 and SUPERQUAD 5.20 software [21,22]. These experimental conditions were previously optimized for the BCS ligand [13] and used in this work. Potentiometric titrations of P1, P2, and P3 as free ligands were carried out. In the case of P3, we only used this method to determine the concentration of the analyzed solution. P1 and P2 systems with 2:1 ligand-to-metal ion molar ratios were analyzed. In Results and Discussion section, nitrogen donors from water molecules were omitted for clarity in all of the formed and described complexes. P3 does not have any anchor group for metal ions in its sequence, therefore, the coordination properties of this ligand were not analyzed.

### 3.3. Spectroscopic Measurements

Visible spectra of complexes for the P1 and P2 systems were recorded at 25 °C on a Varian Cary 50 Bio spectrophotometer (Varian Inc., Palo Alto, CA, USA) in the 300–800 nm range. Circular dichroism and magnetic circular dichroism spectra were recorded on a Jasco J-1500 magnetic circular dichroism spectrometer (Jasco, Japan, Tokyo) in the range of 230–800 nm at 25 °C for all of the synthetic peptides that were analyzed. Spectra were collected at a scan rate speed of 100 nm min^−1^ with a response time of 1 s. A path length of 10 mm was used in both the UV-Vis and CD methods. MCD spectra were measured with the use of a Permanent Magnet PM-491 accessory and collected at a scan rate speed of 100 nm min^−1^ with response time of 1 s, with a 5 mm path length and in a magnetic field of +1.6 T, in the N/S field direction. MCD spectra were also recorded for the previously reported BCS ligand [13], in the same conditions described above. All spectroscopic measurements were collected in the 2.5–10 pH range, in increments of a half unit. The ligand concentration was 7 × 10^−4^ mol/L and solutions were prepared in 0.3 mol/L KCl. Sample volumes were 2.0 mL. The pH values were established using a Mettler Toledo pH-meter by adding small amounts of concentrated KOH and HCl solutions.

## 4. Conclusions

In this paper, the coordination studies of newly designed analogues of somatostatin have been described. All ligands form a series of mononuclear copper(II) complexes. The metal binding starts with only the involvement of imidazole donors present on the side chain of both His residue, and then when the pH is increased, the coordination of amide donors takes place. The presence of the **Phe-Trp-Lys-Thr** motif in the peptide sequence makes the ligand more efficient in metal binding. Moreover, the presence of disulfide bonds in the structure of BCS promotes coordination of the metal ion with donor atoms of the side chain groups, which also affects the efficiency of the coordination process. The presence of a double cycle strongly stiffens and organizes the structure of the molecule, therefore, the donors from the side chain groups are more accessible for the metal ion.

It was also demonstrated that the MCD method is a very promising technology for investigating complex systems containing aromatic amino acid residues.

## Figures and Tables

**Table 1:** Potentiometric data for Cu(II)/P1, Cu(II)/P2 and Cu(II)/BCS systems. P1 and P2 concentrations were 7 × 10^4^ mol/L and pH-metric titration was performed in a 0.3 mol/L KCl solution using sample volumes of 1.5 mL. Measurements were carried out in a 2.5–10 pH range, at 25 °C. The ligands to metal ratio was 2:1 in both analyzed systems.

Species	P1			P2			BCS [13]	
	***logβ ^a^***	***logK ^b^***	logβ* ^c^	***logβ ^a^***	***logK ^b^***	logβ* ^c^	***logβ***	***logK***
Ligands								
HL	10.26 ± 0.02	10.26		10.75 ± 0.05	10.25		10.96	10.96
H_2_L	20.03 ± 0.01	9.78		19.81 ± 0.03	9.62		20.77	9.81
H_3_L	26.61 ± 0.02	6.58		26.38 ± 0.04	6.62		28.02	7.25
H_4_L	32.32 ± 0.02	5.71		31.85 ± 0.04	5.53		33.24	5.22
**Copper(II) Complexes**								
CuH_3_L	-			-			33.14	5.70
CuH_2_L	25.00 ± 0.08		4.97	25.10 ± 0.04		5.29	27.44	7.10
CuHL	-			18.26 ± 0.06	6.79	7.51	20.34	8.82
CuL	10.35 ± 0.11	14.65		10.10 ± 0.07	8.13		11.52	9.54
CuH_−1_L	1.31 ± 0.14	9.04		1.28 ± 0.07	8.84		1.98	20.90
CuH_−3_L	−18.81 ± 0.14	20.12		−18.78 ± 0.07	19.74		−18.92	

^a^ cumulative constants for ligands: L^−^+nH^+^_↔_[H_n_L]^(n−1)+^, for complexes: Cu^2+^ + nH^+^+L^−^_↔_[CuH_n_L]^(n + 1)+^; ^b^ stepwise constants for ligands: [H_n_L]^(n−1)+^+H^+^_↔_[H_n+1_L]^n+^, for complexes: [CuH_n_L]^(n + 1)+^+H^+^_↔_[CuH_n+1_L]^(n + 2)+^; The charges were omitted to facilitate reading. ^c^ corrected constants logβ*_CuHnL_ = log*β_CuHnL_* − log*β_HnL._*

**Table 2:** Spectroscopic data for the Cu(II)/P1 and Cu(II)/P2 systems. The ligand concentrations were 7 × 10^−4^ mol/L and pH-metric titrations were performed in a 0.3 mol/L KCl solution using samples volumes of 2 mL. Measurements were carried out in a 2.5–10 pH range, at 25 °C. The ligand to metal ratios was 2:1 in both analyzed systems.

	UV-Vis	CD	
Dominating Complex	λ [nm]	λ [nm]	Δε [M^−1^ cm^−1^]
P1. *(Ac-c(-S-Cys-Pro-His^a^-Lys-Lys-His^b^-Pro-Cys-S-)-NH_2_)*			
CuH_2_L	665	-	-
CuL	592 510	623 ^a^ 482 ^a^ 314 ^b^ 254 ^c^	0.211 −0.873 0.861 5.14
CuH_−1_L	580 ^sh^ 506	617 ^a^ 482 ^a^ 315 ^b^ 254 ^c^	0.782 −1.43 1.45 4.43
CuH_−3_L	580 ^sh^ 506	617 ^a^ 482 ^a^ 315 ^b^ 254 ^c^	0.899 −1.43 1.45 4.19
P2. *(c(Ser-Pro-His^a^-Lys-Lys-His^b^-Pro-Ser-Phe-Trp-Lys-Thr))*			
CuH_2_L	658	-	-
CuHL	609	624 ^a^ 486 ^a^ 323 ^b^ 260 ^c^	0.133 −0.325 0.289 0.935
CuL	606 ^sh^ 499	619 ^a^ 493 ^a^ 320 ^b^ 259 ^c^	0.487 −0.721 0.633 2.56
CuH_−1_L	606 ^sh^ 497	626 ^a^ 492 ^a^ 320 ^b^ 258 ^c^	0.689 −1.07 0.904 3.133

^a^ d-d transition, ^b^ N_amide_ → Cu(II) CT, ^c^ N_Im_ → Cu(II) CT, ^sh^ shoulder.

## Abbreviations

BCS	Bicyclic Somatostatin analogue
CD	Circular Dichroism spectroscopy
CT	Charge Transfer
Dde	N-1-(4,4-dimethyl-2,6-dioxocyclohex-1-ylidene)ethyl group
DIC	N,N′-Diisopropylcarbodiimide
DIPEA	N,N-Diisopropylethylamine
DMF	Dimethylformamide
MCD	Magnetic Circular Dichroism Spectroscopy
MW	Microwave
SST	Somatostatin
TFA	Trifluoroacetic acid
TIS	Triisopropyl silane
